# The added value of radiomics from dual-energy spectral CT derived iodine-based material decomposition images in predicting histological grade of gastric cancer

**DOI:** 10.1186/s12880-022-00899-y

**Published:** 2022-10-03

**Authors:** Cen Shi, Yixing Yu, Jiulong Yan, Chunhong Hu

**Affiliations:** 1 Department of Radiology, The First Affiliated Hospital of Soochow University, No. 188, Shizi Street, Suzhou, 215006 Jiangsu People’s Republic of China; 2 Institute of Medical Imaging of Soochow University, No. 188, Shizi Street, Suzhou, 215006 Jiangsu People’s Republic of China

**Keywords:** Gastric cancer, Dual-energy spectral CT, Iodine-based material decomposition images, Radiomics, Histologic grade

## Abstract

**Background:**

The histological differentiation grades of gastric cancer (GC) are closely related to treatment choices and prognostic evaluation. Radiomics from dual-energy spectral CT (DESCT) derived iodine-based material decomposition (IMD) images may have the potential to reflect histological grades.

**Methods:**

A total of 103 patients with pathologically proven GC (low-grade in 40 patients and high-grade in 63 patients) who underwent preoperative DESCT were enrolled in our study. Radiomic features were extracted from conventional polychromatic (CP) images and IMD images, respectively. Three radiomic predictive models (model-CP, model-IMD, and model-CP–IMD) based on solely CP selected features, IMD selected features and CP coupled with IMD selected features were constructed. The clinicopathological data of the enrolled patients were analyzed. Then, we built a combined model (model-Combine) developed with CP–IMD and clinical features. The performance of these models was evaluated and compared.

**Results:**

Model-CP–IMD achieved better AUC results than both model-CP and model-IMD in both cohorts. Model-Combine, which combined CP–IMD radiomic features, pT stage, and pN stage, yielded the highest AUC values of 0.910 and 0.912 in the training and testing cohorts, respectively. Model-CP–IMD and model-Combine outperformed model-CP according to decision curve analysis.

**Conclusion:**

DESCT-based radiomics models showed reliable diagnostic performance in predicting GC histologic differentiation grade. The radiomic features extracted from IMD images showed great promise in terms of enhancing diagnostic performance.

## Introduction

Gastric cancer (GC) remains one of the most aggressive digestive system malignancies, with over one million new cases expected in 2020 and an estimated 769,000 deaths, ranking fifth in incidence and fourth in fatality globally [1]. TNM (tumor, lymph node, and metastatic) staging was shown to be substantially correlated with the 5-year survival rate of GCs. However, clinical results among individuals getting comparable therapy at the same TNM stage differed significantly [2]. A growing number of studies have found that different histological differentiation types are closely related to the prognosis of GCs. According to the Japanese Gastric Cancer Classification and Lauren’s Classification, GC is histologically categorized into differentiated type, expanding, or intestinal type, and undifferentiated type, or diffuse type [3, 4]. The undifferentiated type of GC corresponds to poorly differentiated gastric carcinoma in the World Health Organization Classification and encompasses a variety of subtypes, such as signet ring cell carcinoma (SRCC) and non-SRCC (NSRCC). Gastric cancers of the undifferentiated type are known to have a higher rate of lymph node metastases and a worse prognosis than those of well differentiated type [5, 6]. As a result, the histologic differentiation type has come to be viewed as a crucial factor in assessing tumor progression, treatment options, and predicting outcomes in GC patients [7, 8]. Therefore, accurate classification and risk stratification of GC patients is critical for making management decisions and predicting prognosis at the time of diagnosis.

Currently, endoscopic biopsy specimens are utilized to determine the histologic type and grade of gastric cancer prior to surgery; however, they are insufficient to represent the complete tumor due to intratumorally heterogeneity [9–11]. Furthermore, as an invasive procedure, endoscopic biopsy carries a risk of bleeding, perforation, and infection afterward [12, 13]. As a result, an alternate, noninvasive method for determining GC histologic type preoperatively is needed to enhance the currently utilized method.

Multi-detector computed tomography (MDCT) was extensively employed for preoperative GC staging because of its wide availability and accessibility. Tsurumaru et al. investigated the enhancement patterns of different histologic types on dynamic contrast-enhanced CT [8]. However, the enhancement pattern may change owing to the tumor’s heterogeneous sections, which may also be influenced by the CT machine used and the scanning time. According to Lee et al., perfusion CT parameters can help in the preoperative diagnosis of poorly cohesive carcinoma [14]. Perfusion CT, on the other hand, is time-consuming, requires patients’ cooperation, and exposes patients to high radiation doses, hence it is rarely routinely used in GC clinical examination. Dual-energy spectral CT (DESCT) was seen as a possible advancement in CT that offer extra information such as material decomposition (MD) images (e.g., iodine- or water-based MD images), and monochromatic images. A previous study evaluated the effectiveness of DESCT in discriminating histological types of GC [15]. However, this study only analyzed the role of iodine concentration (IC) in GC, and their region of interests (ROIs) were drawn in round shapes on 2D images, implying that the diagnostic usefulness of DESCT had not been fully assessed.

Radiomics is the process of extracting quantitative information from radiological images using high-throughput analysis and choosing features to build a signature for comprehensive understanding of tumors’ characterization [16]. These signatures can be employed alone or in combination with other patient-related data (e.g., clinical data; pathological data) to improve tumor phenotyping, treatment response prediction, and prognosis evaluation [17]. Radiomics has been found superior in predicting tumor invasion depth, lymph node metastasis, and assessing GC response to neoadjuvant chemotherapy [18–20]. Although radiomics signatures have been used to predict the histological status of GC in earlier studies [21, 22], the diagnostic utility of radiomics features combined with dual-energy signatures and clinical features is unknown.

In this study, we aimed to explore the prediction performance of DESCT-derived radiomics signatures, combined with iodine-based MD images features and clinical features, in predicting the histologic differentiation grade of GC preoperatively.

## Material and methods

The study was a retrospective study and was approved by our Hospital Medical Ethics Committee. Informed consent was waived. The study was performed in accordance with the Declaration of Helsinki. All methods were carried out in accordance with relevant guidelines and regulations.

### Patients

**Fig. 1:**
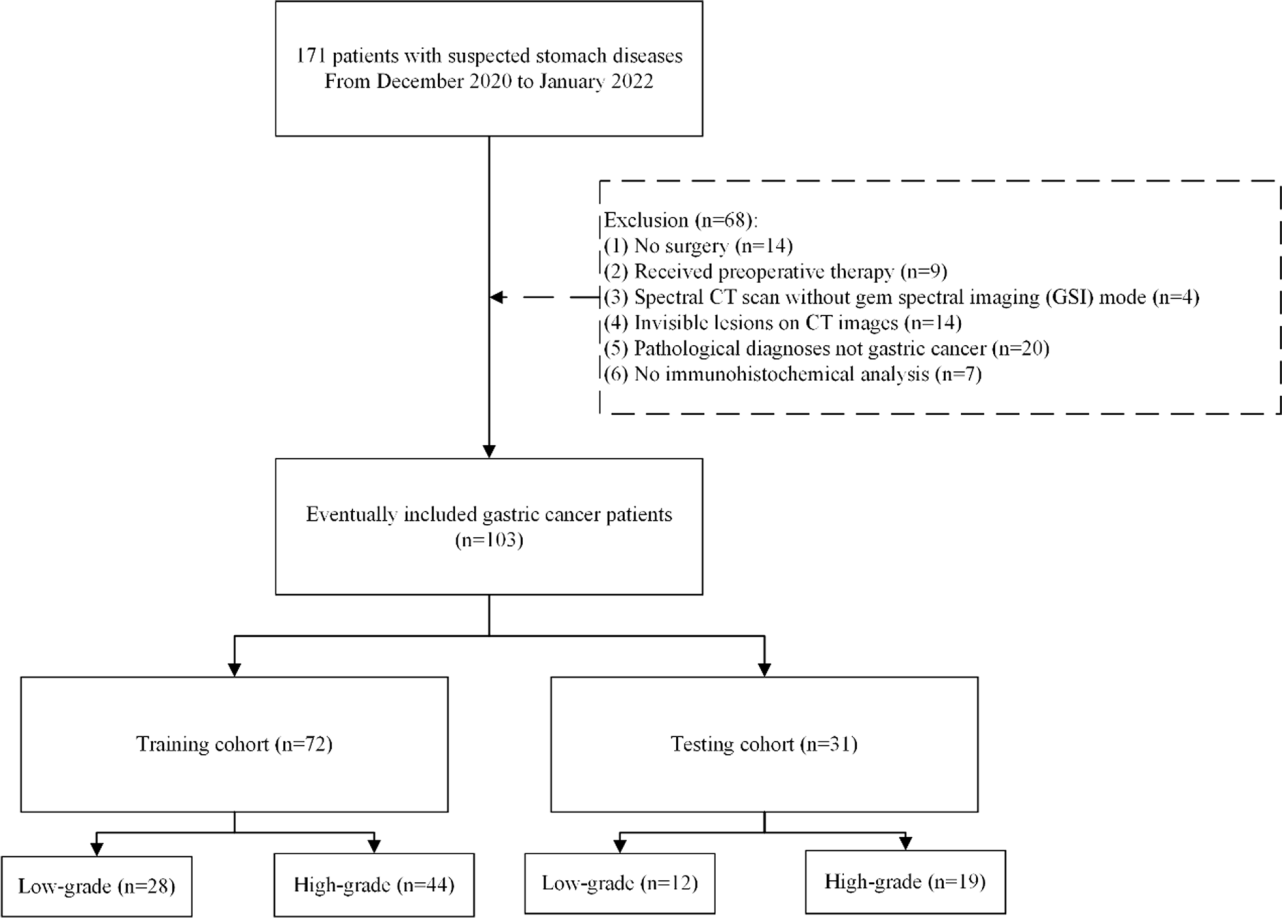
Flowchart of patient enrollment

From December 2020 to January 2022, 171 patients with probable stomach diseases were scanned using a dual-energy multi-detector row CT before surgery. The following were the study’s inclusion criteria: (1) no preoperative neoadjuvant chemotherapy or radiotherapy; (2) contrast-enhanced CT examination within two weeks of surgery; (3) contrast-enhanced CT scan with gemstone spectral imaging (GSI) mode; (4) visible tumor lesions on CT images; (5) specimen with pathologic diagnosis of gastric cancer; (6) specimen with pathologic diagnosis of histologic differentiation grade. Finally, 103 patients were enrolled in our research (Fig. 1). Clinical data, including age, gender, tumor location, tumor size, pT stage, pN stage, and preoperative tumor makers including carcinoembryonic antigen level (CEA, normal reference value: < 5 ng/ml), alpha fetoprotein (AFP, normal reference value: < 8.78 µg/L), carbohydrate antigen 125 (CA125, normal reference value: < 35U/ml), carbohydrate antigen (CA19-9, normal reference value: < 37U/ml), carbohydrate antigen 153 (CA153, normal reference value: < 31.3U/ml), carbohydrate antigen 72-4 (CA72-4, normal reference value: < 6U/ml) were obtained by evaluating the medical records for analysis.

### Histopathological examination

Two pathologists blinded to the CT data evaluated each tumor specimen. Tissue sections were stained with a hematoxylin and eosin (HE) stain according to standard procedures. The pathologic T and N stages according to the American Joint Committee on Cancer (AJCC) staging system (8th edition) [23], tumor differentiation status, and tumor long axis were all documented. The histologic differentiation results were divided into low- and high-grade groups in accordance with the 5th edition of the WHO tumor classification of the digestive system and Japanese Gastric Cancer Classification [3, 24]. Well and moderately differentiated adenocarcinomas and papillary adenocarcinomas are classified as low-grade, whereas poorly differentiated adenocarcinomas, signet ring cell carcinoma, or mucinous adenocarcinoma are high-grade.

### CT Imaging

After a night of fasting to empty the stomach, all patients underwent CT scans. To allow for stomach distention, all patients were instructed to drink 800–1000 ml of tap water. All stomach CT scans were performed using the same scan protocol on a high-definition dual-energy spectral CT scanner (Discovery CT750HD, GE Healthcare, Wisconsin, USA). All the patients were positioned in a supine position on the scanner, and a routine non-enhanced CT scan was done first. The contrast-enhanced scans were then completed utilizing a single tube, rapid dual kVp (80 kVp and 140 kVp) switching technique (GSI mode). Other scan parameters included a 5 mm collimation thickness with 40 mm detector coverage, 600 mA tube current, 0.984 helical pitch, and 0.6 s rotation speed. Contrast media (Ultravist370; Schering, Berlin, Germany) with a standard dose (1.5 ml/kg of body weight) was injected at a flow rate of 4 ml/s with a power injector (Urich REF XD 2060-Touch, Germany). After the start of the contrast injection, two phase-enhanced CT images, comprising the arterial phase and portal phase, were obtained, encompassing the whole abdomen and pelvis, respectively. Arterial phase scanning began 10 s after the trigger threshold (100 HU) was reached at the level of the supra-celiac abdominal aorta. Portal phase scanning was started at 60 s delays after the injection.

The CT images were reconstructed by using projection-based material-decomposition software. To balance image noise and spatial resolution, the reconstruction thickness was 1.25 mm, with 1.25 mm intervals. The adaptive statistical iterative reconstruction (ASIR) algorithm was employed to reduce image noise on the decomposition images. The percentage of ASIR was 30%. After scanning, conventional polychromatic images were generated. In addition, iodine-based MD images of the portal phase were reconstructed using the GSI Volume Viewer software package at the ADW 4.7 workstation (GE Healthcare, Milwaukee, WI, USA). The conventional polychromatic images and iodine-based MD images were ultimately used for analysis.

### Tumor segmentation

Because most gastric cancer lesions showed considerable enhancement and could be clearly separated from the neighboring tissues in the portal phase, the portal phase CT images were collected for further tumor segmentation [25]. The radiologists were advised of the verified surgical locations of the tumor but were blinded to other clinical information and pathologic outcomes when completing the segmentation because our study did not attempt to assess the detection capabilities of CT radiomics analysis. The tumors were segmented using 3D Slicer 4.11.2 software (www.slicer.org) by a radiologist (reader 1) with 10 years of experience in CT abdominal imaging.

**Fig. 2:**
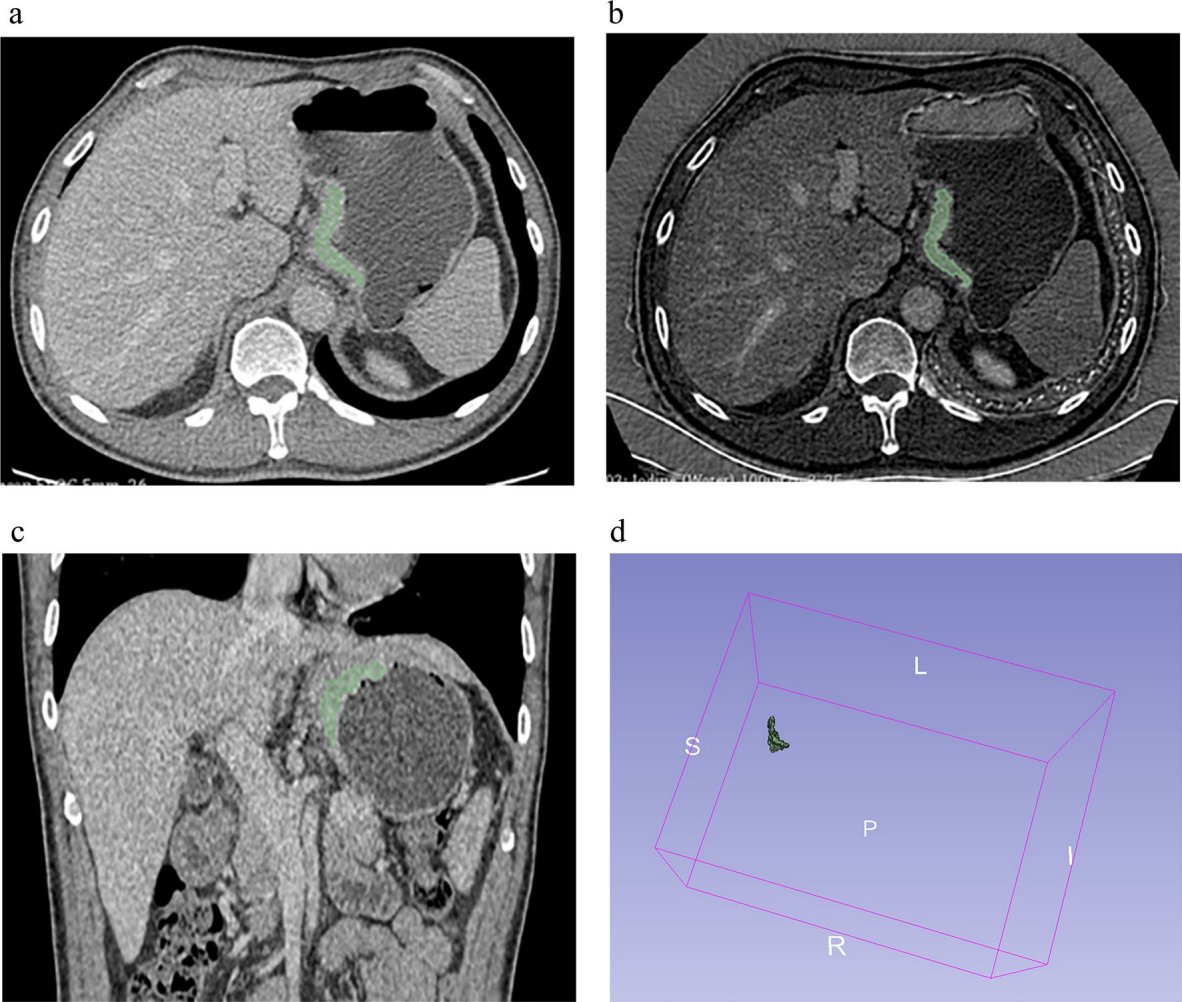
Delineation of the volume of interest (VOI). A gastric cancer located in the cardia was shown in **a** axial portal phase images; **b** iodine-based MD images; **c** coronal multiplanar reconstruction images. **d** Three-dimensional VOI of the tumor was displayed

Initially, conventional polychromatic images were used to identify the volume of interest (VOI) for the whole tumor. All lesions had regions of interest (ROIs) drawn slice-by-slice on the 2D images, with the contour drawn slightly within the margins of the tumor masses to avoid including adjacent air or fat. The corresponding sagittal and coronal planes of the tumors might be utilized as a reference if the lesion was difficult to recognize in the axial plane. The ROIs were then used to reconstruct VOIs. The resulting 3D segmentation was then replicated onto the geometrically identical images of the IMD images. Figure 2 depicts a manual segmentation example in action.

### Feature extraction

The Slicer Radiomics extension, which integrates the PyRadiomics library into 3D Slicer [26], was used to extract features. The images was preprocessed in the following way to facilitate consistent feature extraction: spatial resampling to 1 × 1 × 1 mm^3^; intensity discretization to a fixed bin width of 25. Radiomics features were calculated on the preprocessed images using the wavelet and Laplacian of Gaussian (LoG) filters with varying λ-parameters (= 1.0, 2.0, 3.0, 4.0, 5.0). In total, 1316 radiomic features were extracted, including (1) first-order statistics features; (2) shape features; (3) texture features, including gray level co-occurrence matrix (GLCM), gray level size zone matrix (GLSZM), gray level run length matrix (GLRLM), gray level dependence matrix (GLDM), and neighboring gray tone dependence matrix (NGTDM); (4) statistical features produced from LoG filtered domains; (5) wavelet features derived from wavelet filtered domains.

A set of 30 lesions was chosen at random to assess the repeatability of radiomics features, and two radiologists (reader 1; reader 2, with 12 years of work experience) completed individual segmentation repeats. Following that, the intraclass and interclass correlation coefficients (ICC) were calculated. Features with ICCs of 0.80 or above (both inter- and intra-observer classes) were deemed reliable and selected for further investigation.

### Features selection and model construction

**Fig. 3:**
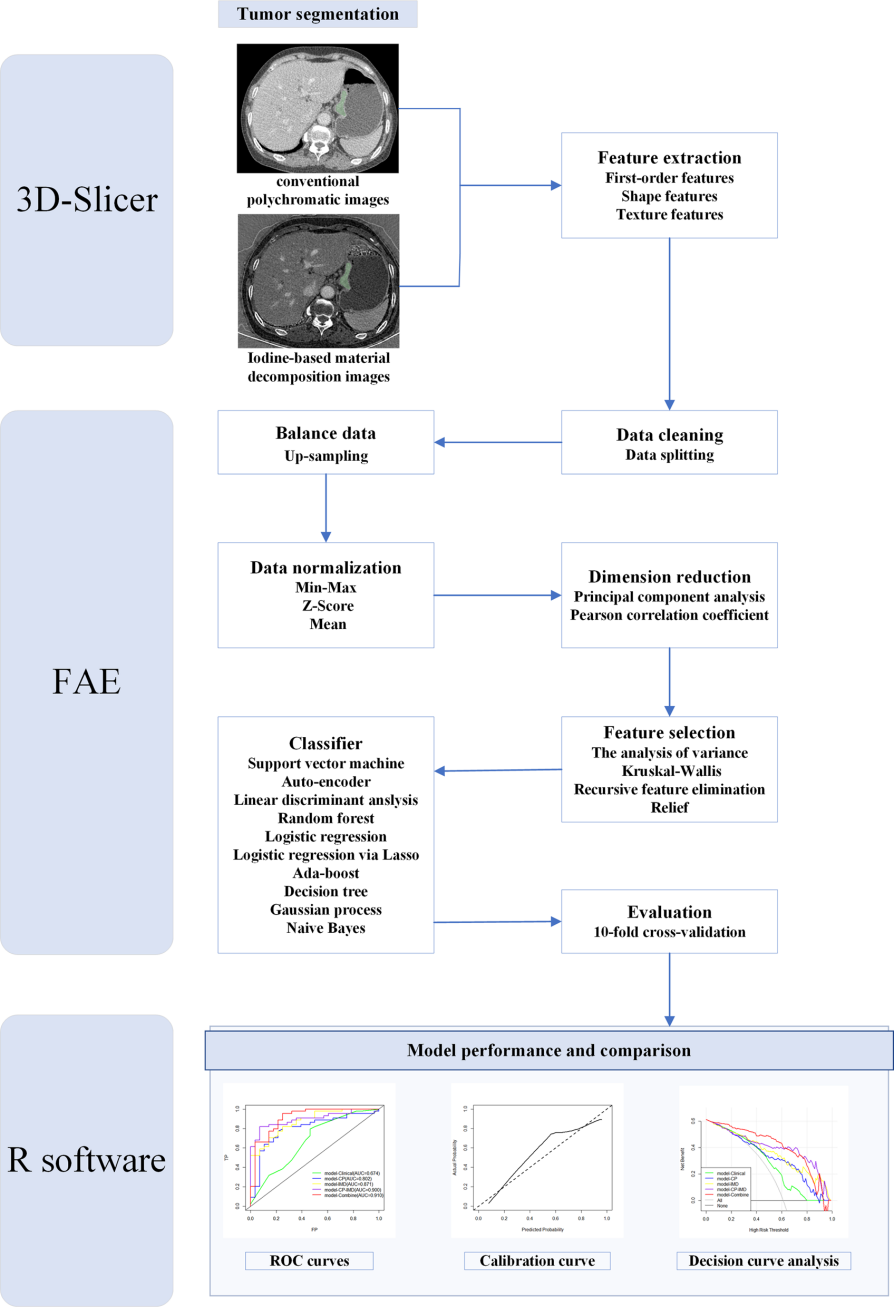
Flowchart of our study

For model establishment and assessment, all enrolled patients were randomly divided into training and testing cohorts at a ratio of 7:3 (72 and 31 patients, respectively). An open-source free application, FeAture Explorer Pro (FAE, version 0.5.0; https://github.com/salan668/FAE) on Python(3.7.6) [27], was used to analyze and assess all of the radiomics models. To remove the unbalance of the training data set, we used up-sampling method to make positive/negative samples balance by repeating random low-grade cases. Data normalization (three approaches), dimension reduction (two ways), feature selection algorithms (four methods, 20 features), and classifier development (ten methods) were then used to construct the radiomics models. All conceivable combinations of approaches were used to build radiomics models to give additional options for model development and to select more appropriate modeling methods. The procedure for model development is shown in Fig. 3.

Models based on the conventional polychromatic images features (model-CP), iodine-based MD images features (model-IMD), and a model combining conventional polychromatic images and iodine-based MD images features (model-CP–IMD) were developed. In addition, the radiomics signature and independent clinicopathologic factors with P values less than 0.05 in the univariable analysis were merged to form a combined prediction model (model-Combine). Ten-fold cross-validation (CV) was applied to the training cohort to determine the candidate combinations of the selected features and classifiers. The cross-validation was then performed on the entire training set to determine the candidate combination, following which the final model was built by all training cases and evaluated on the test cohort. The area under the receiver operator characteristics curve (AUC) of the classification results was calculated for each tested condition. The radiomics model with the greatest performance in the cross-validation set would be picked as the final model. When several models had similar or almost identical prediction performance, the one with the fewest features was picked to reduce the model’s complexity and the risk of non-generalization. At a cutoff value that maximized the value of the Youden index, the accuracy, sensitivity, specificity, positive predictive value (PPV), and negative predictive value (NPV) were calculated.

### Model performance and comparison

The receiver operating characteristic curve (ROC) was used to evaluate the discrimination performance of the aforementioned four models, and AUC was compared using DeLong’s test. In both training and testing cohorts, calibration curves, Hosmer–Lemeshow test, and Brier score were employed to assess the model’s goodness of fit. Furthermore, decision curve analysis (DCA) was conducted to evaluate the model’s clinical applicability by measuring the net benefits at different threshold probabilities. Figure 3 shows the flowchart of our study.

### Statistical analysis

The differences in clinical characteristics between the two cohorts were assessed using an independent samples t-test or Mann–Whitney U-test, depending on whether they were normal distribution (Kolmogorov–Smirnov test). The difference between category variables was assessed with chi-square test or Fisher exact test. All P values were two-sided, and P < 0.05 was considered statistically significant. Model performance assessments were performed using R software (version 4.1.2, http://www.rproject.org). Other statistical analyses were carried out using IBM SPSS Statistics (Version 21; IBM Corp., New York, USA).

## Results

### Clinical characteristics

The clinical data of the study cohort patients were presented in Table 1. Among all 103 patients, low-grade was pathologically diagnosed in 40 patients, and high-grade was pathologically diagnosed in 63 patients. All the clinical factors, including age, gender, tumor location, tumor size, and all tumor makers were not significantly associated with tumor differentiation grade except pT and pN stage (all P-values < 0.05 in training and testing cohorts). We constructed a clinical model based on the pT and pN stage, and its predictive performance was shown in Table 2.

**Table 1:** Clinical characteristics of the patients in training and testing cohorts

Characteristics	Training set (n = 72)			Testing set (n = 31)		
	Low-grade (n = 28)	High-grade (n = 44)	P value	Low-grade (n = 12)	High-grade (n = 19)	P value
Age	70.29 ± 8.19	66.43 ± 11.12	0.119	65.33 ± 11.48	64.11 ± 11.72	0.777
Gender			0.714			0.178
Female	9	16		1	7	
Male	19	28		11	12	
Tumor long axis	4.53 ± 2.73	5.39 ± 2.85	0.209	3.60 ± 1.71	5.66 ± 3.55	0.039
pT stage			0.005			0.025
T1	5	0		5	0	
T2	4	5		0	1	
T3	5	4		2	4	
T4	14	35		5	14	
pN stage			< 0.001			< 0.001
N0	11	6		6	1	
N1	5	2		2	1	
N2	7	17		3	4	
N3	5	29		1	13	
Location			0.115			0.278
Upper 1/3	10	10		3	4	
Middle 1/3	10	14		5	5	
Lower 1/3	7	16		4	7	
Multiple	1	4		0	3	
AFP			1.000			1.000
Normal	26	41		12	19	
Abnormal	2	3		0	0	
CEA			0.147			1.000
Normal	23	42		12	19	
Abnormal	5	2		0	0	
CA125			0.884			0.510
Normal	25	41		12	17	
Abnormal	3	3		0	2	
CA19-9			0.553			0.409
Normal	22	37		7	15	
Abnormal	6	7		5	4	
CA72-4			0.620			0.510
Normal	23	34		12	17	
Abnormal	5	10		0	2	
CA153			1.000			1.000
Normal	27	43		12	19	
Abnormal	1	1		0	0

**Table 2:** Diagnostic performance of models in training and testing cohorts

	AUC (95% CI)	ACC	SEN	SPE	PPV	NPV
Model-Clinical						
Training	0.674 (0.543–0.804)	0.694	0.795	0.536	0.729	0.625
Testing	0.847 (0.612–0.950)	0.710	0.579	0.917	0.917	0.579
Model-CP						
Training	0.802 (0.693–0.911)	0.792	0.818	0.750	0.837	0.724
Testing	0.781 (0.612–0.950)	0.742	0.737	0.750	0.824	0.643
Model-IMD						
Training	0.871 (0.793–0.950)	0.792	0.818	0.750	0.837	0.724
Testing	0.759 (0.582–0.936)	0.774	0.790	0.750	0.833	0.692
Model-CP–IMD						
Training	0.900 (0.830–0.971)	0.861	0.818	0.927	0.947	0.765
Testing	0.851 (0.711–0.991)	0.839	0.842	0.833	0.889	0.769
Model-Combine						
Training	0.910 (0.837–0.983)	0.875	0.955	0.750	0.857	0.913
Testing	0.912 (0.778–1.000)	0.936	0.747	0.917	0.974	0.917

*AUC* area under the receiver operating curve, *95% CI* 95% confidence interval, *ACC* accuracy, *SEN* sensitivity, *SPE* specificity, *PPV* positive predictive value, *NPV* negative predictive value

### Radiomic features and model establishment

**Fig. 4:**
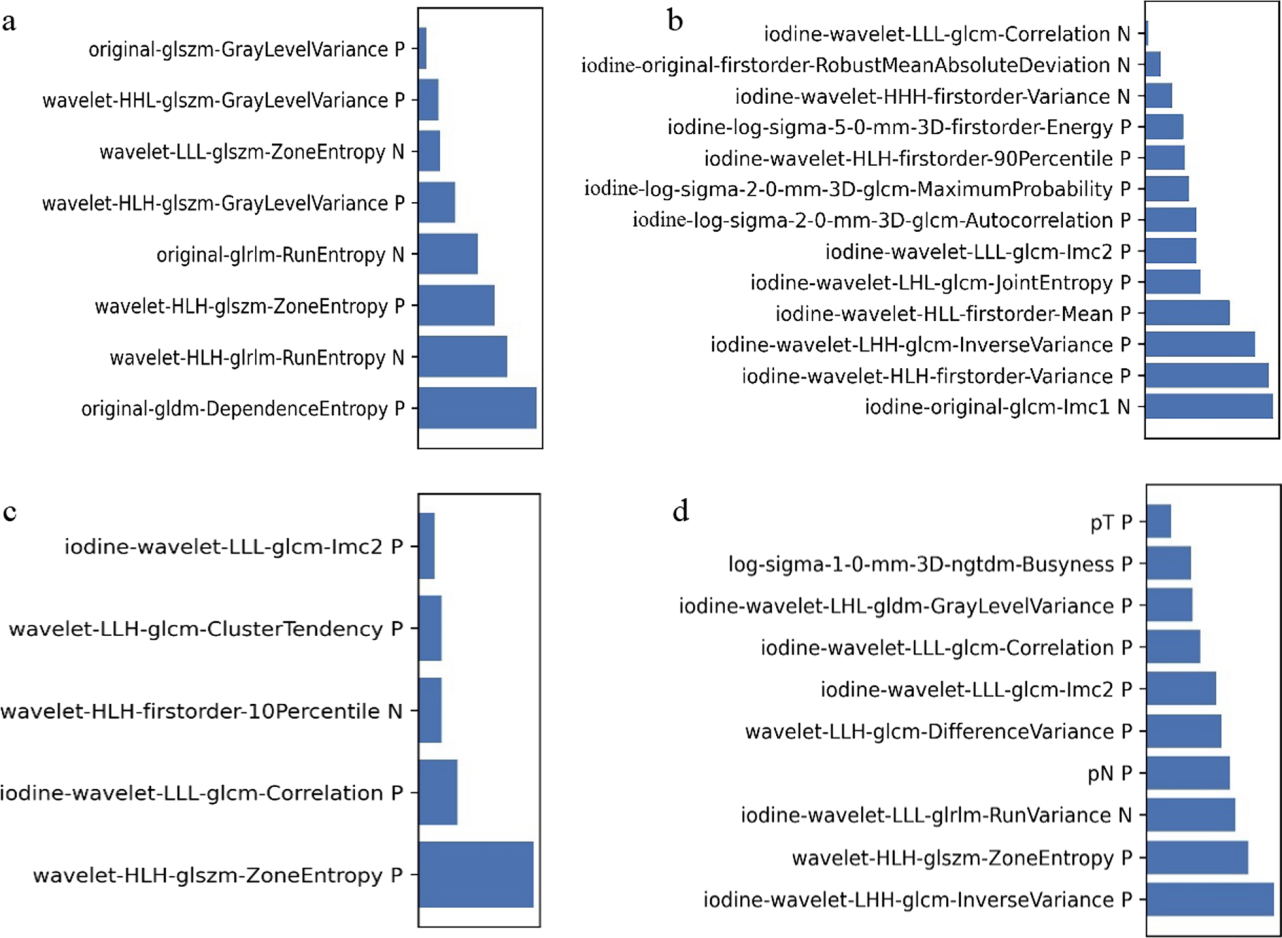
Features contained in models and their weights. **a** Model-CP; **b** model-IMD; **c** model-CP–IMD; **d** model-Combine

After consistency, a total of 898 features from CP images and 484 features from IMD images were selected for further analysis. For model-CP, the pipeline using Mean data normalization, Pearson Correlation Coefficient (PCC) dimension reduction, Kruskal–Wallis (KW) feature selector, and Linear Discriminant Analysis (LDA) classifier yielded the highest AUC using 8 features. For model-IMD, the pipeline using Z-score data normalization, PCC dimension reduction, recursive feature elimination (RFE) feature selector, and auto-encoder (AE) classifier yielded the highest AUC using 13 features. For model-CP–IMD, the pipeline using Z-score data normalization, PCC dimension reduction, RFE feature selector, and Gaussian process (GP) classifier yielded the highest AUC using 5 features. For model-Combine, the pipeline using Mean data normalization, PCC dimension reduction, RFE feature selector, and support vector machine (SVM) classifier yielded the highest AUC using 10 features. The selected features were shown in Fig. 4.

### Comparison of the predictive performance among models in training and testing cohorts

**Fig. 5:**
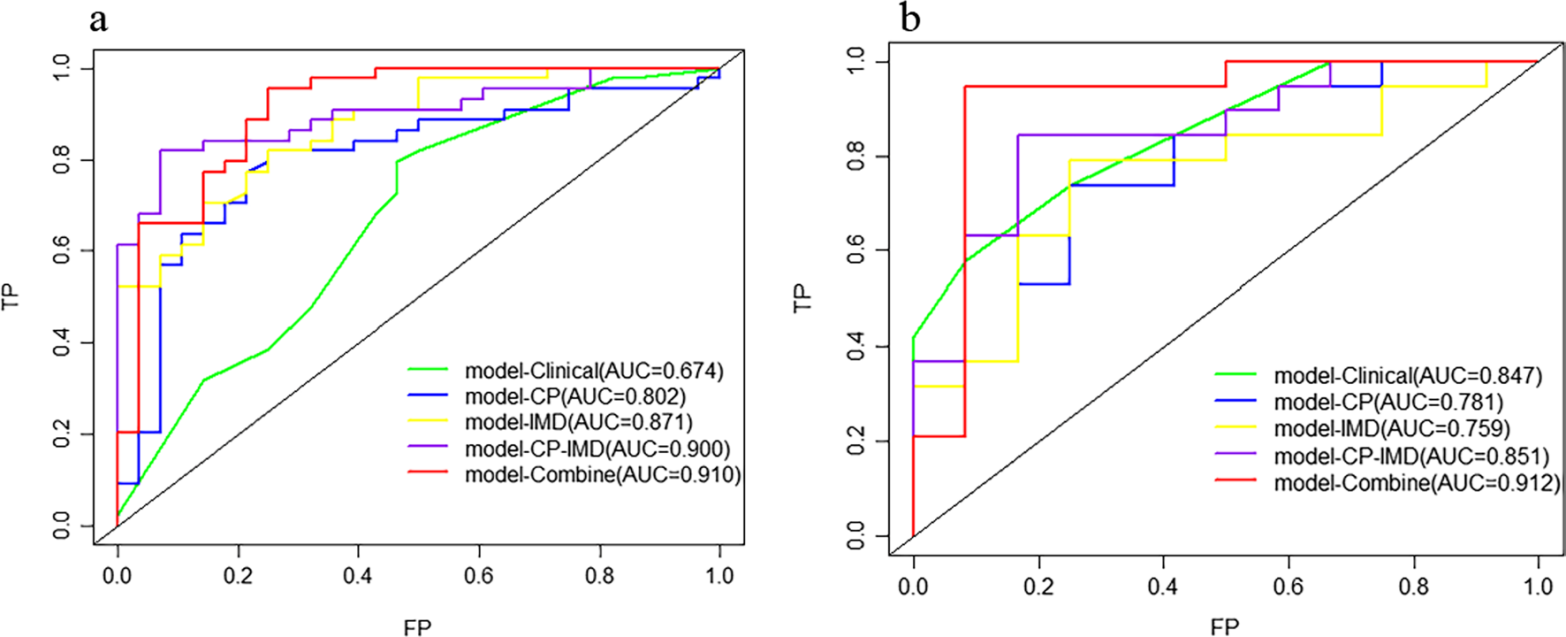
ROC curves of the models in **a** training set and **b** testing set

**Fig. 6:**
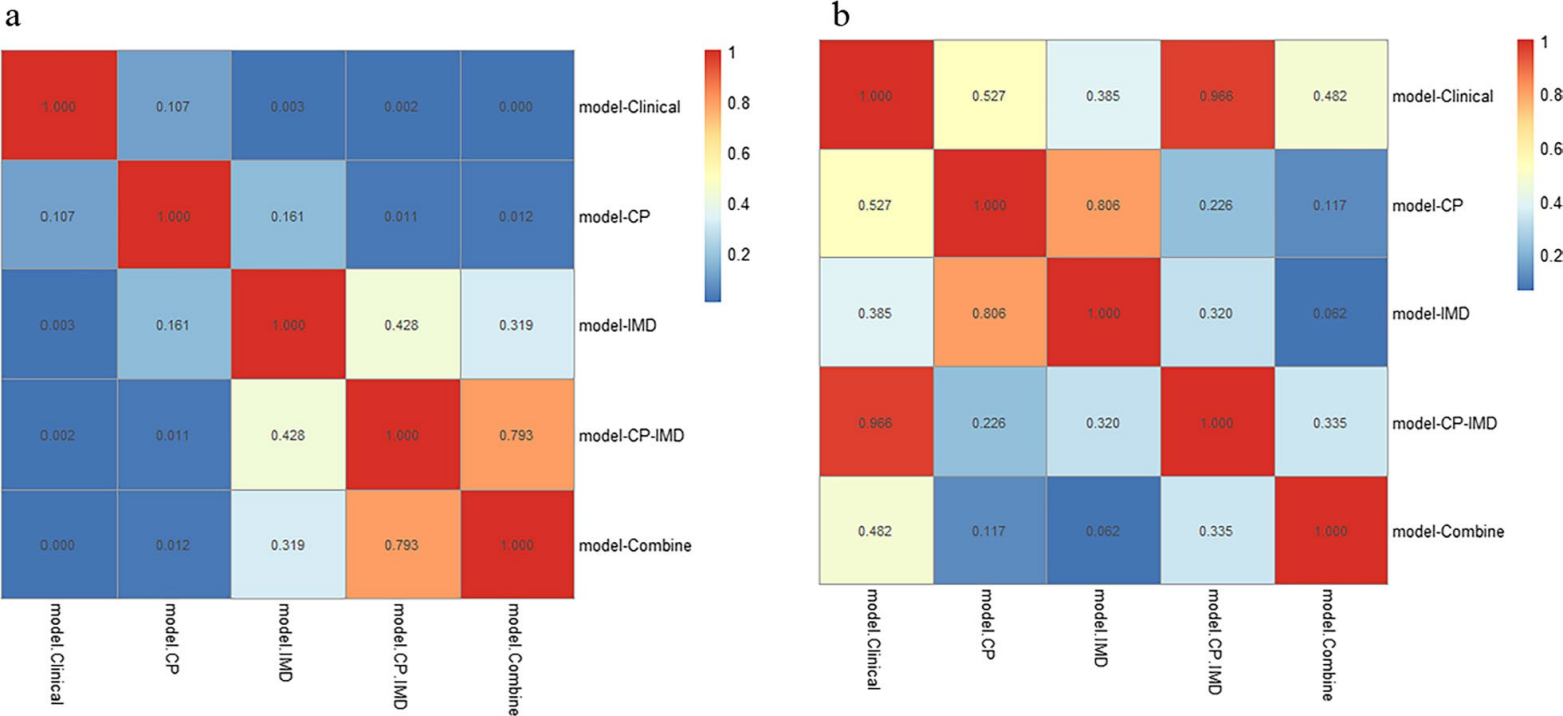
DeLong’s test results in **a** training set and **b** testing set

The ROC curves of all models are shown in Fig. 5. The AUC values and statistical values, including accuracy, sensitivity, specificity, PPV, and NPV, are demonstrated in Table 2. In the training set, model-Clinical reached an AUC of 0.674 (95% CI: 0.543–0.804). Model-CP and model-IMD reached an AUC of 0.802 (95% CI: 0.693–0.911) and 0.871 (95% CI: 0.793–0.950), respectively. Model-CP–IMD revealed some improvement, with an AUC of 0.900 (95% CI: 0.830–0.971). In addition, model-Combine developed combined with conventional polychromatic images features, iodine-based images features, pT stage, and pN stage, reached the highest AUC of 0.910 (95% CI: 0.837–0.983). Model-CP–IMD and model-Combine showed significantly better performance than model-CP (P = 0.011 and 0.012). Model-IMD, model-CP–IMD, and model-Combine all showed significantly better performance than model-Clinical (all P-values < 0.05). There was no significant difference between other models (all P-values > 0.05). For the testing set, model-Combine also demonstrated better performance for predicting histologic grade (AUC = 0.912, 95% CI: 0.778–1.000) than other models. Model-CP–IMD (AUC = 0.851, 95% CI: 0.711–0.991) reached higher AUC than model-Clinical, model-CP, and model-IMD (AUC = 0.847 (95% CI: 0.612–0.950), AUC = 0.781 (95% CI: 0.612–0.950), and AUC = 0.759 (95% CI: 0.582–0.936), respectively), Delong’s test showed there was no significant difference between all the models (all P-values > 0.05). Delong’s test results were shown in Fig. 6.

**Fig. 7:**
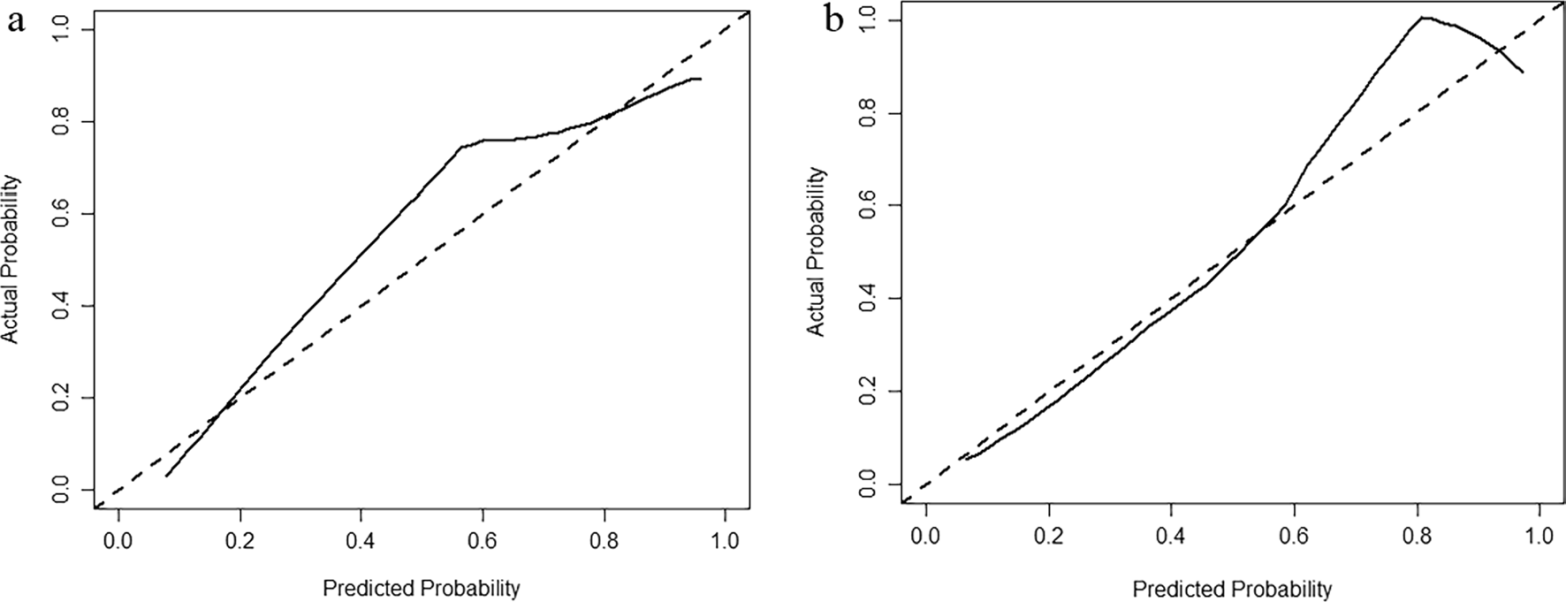
Calibration curves of model-Combine in **a **training cohort and **b** testing cohort

**Fig. 8:**
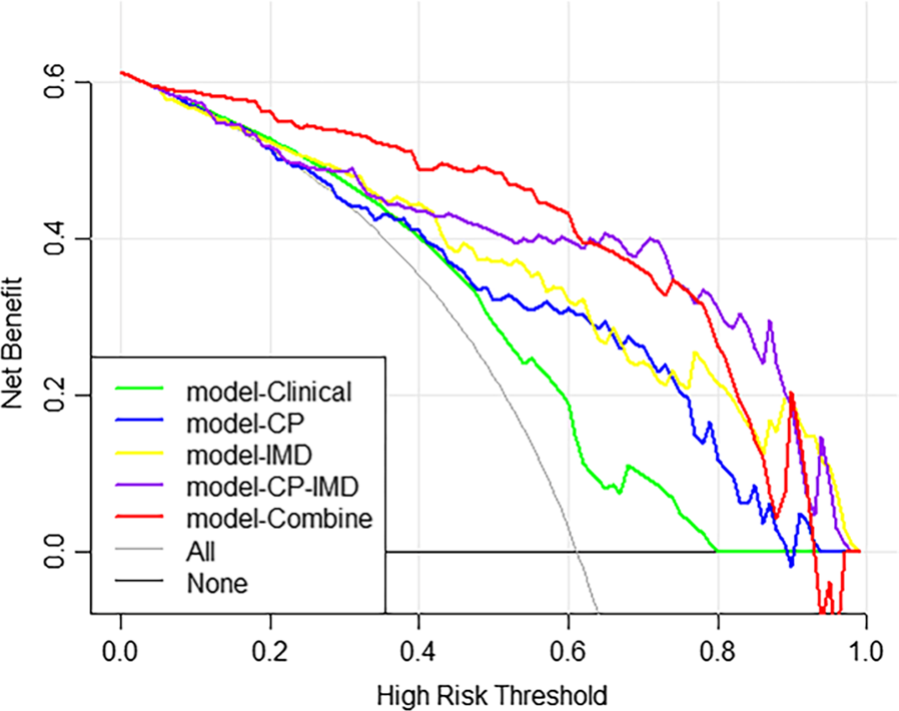
Decision curve analysis for all models in the whole dataset. A larger area under the decision curve indicates a better clinical utility. Model-Combine added more net benefit than model-CP at the range of 0.1–0.9 and model-CP–IMD added more net benefit than model-CP at the range of 0.3–1.0. In comparison to model-Clinical, model-Combine owned a larger net benefit at a range threshold probability of 0.05–0.95

The Hosmer–Lemeshow test showed a statistically favorable calibration of all the models in both cohorts (P = 0.593, 0.431, 0.700, 0.600, and 0.148, respectively for model-Clinical, model-CP, model-IMD, model-CP–IMD, and model-Combine in the training set, and P = 0.933, 0.598, 0.146, 0.182, and 0.378, respectively for model-CP, model-IMD, model-CP–IMD, and model-Combine in the testing set). According to the calibration curves and Brier score, model-CP–IMD and model-Combine showed better goodness of fit than model-CP and model-IMD in both training and testing sets (Brier score = 0.210, 0.173, 0.141, 0.126, and 0.115, respectively for model-Clinical, model-CP, model-IMD, model-CP–IMD, and model-Combine in the training set; Brier score = 0.146, 0.184, 0.193, 0.158, and 0.086, respectively for model-CP, model-IMD, model-CP–IMD, and model-Combine in the testing set). The calibration curves of model-Combine showed a good agreement between predicted and actual events in both cohorts (Fig. 7). In terms of the clinical gain, DCA illustrated that model-Combine added more net benefit than model-CP at a range of 0.1–0.9 and model-CP–IMD added more net benefit than model-CP at a range of 0.3–1.0. In comparison to model-Clinical, model-Combine owned a larger net benefit at a range threshold probability of 0.05–0.95, indicating better clinical utility (Fig. 8).

## Discussion

The radiomics models of conventional polychromatic images and iodine-based MD images were established in this work to predict histological grade in GC patients, and results showed that they had comparative performance (AUC = 0.781 and 0.759, accuracy = 0.742 and 0.774 for model-CP and model-IMD, respectively). We then integrated these two types of signatures to create a combined radiomics model (model-CP–IMD) for predicting the histologic differentiation type of GC prior to surgery. The model-CP–IMD performed better, with a higher AUC value of 0.851 and a higher accuracy value of 0.839, reflecting the discrimination value of iodine-based MD images from DESCT. In addition, with an AUC value of 0.912, model-Combine, which incorporated radiomic and clinical characteristics, outperformed other models in predicting the histologic differentiation type of GC.

An accurate diagnosis of the histologic differentiation type of GC is required since it affects therapy options and patients’ prognosis [28]. Biopsies are taken from the surface of the lesion, posing the risk of bleeding or infection, and may not exactly correspond to the final specimen from gastrectomy or endoscopic resection [11, 12, 29]. As a result, a noninvasive approach for precisely predicting the histologic differentiation grade of GC prior to surgery is required. By extracting high-throughput quantitative imaging features, radiomics had previously demonstrated their efficacy in discriminating histologic type preoperatively. Li et al. revealed that whole-tumor-based histogram and texture analysis using intravoxel incoherent motion were able to distinguish pathologic subtypes of GC with an AUC of 0.948 [30]. While CT is more widely used and serves as the standard-of-care imaging method for the preoperative evaluation of GC, building radiomics models using features derived from routinely acquired contrast CT images could be more convenient and efficient.

Liu et al. only used CT texture parameters to distinguish poorly differentiated GCs, yielding an AUC of 0.774 [21]. The work carried out by Huang et al. established a nomogram based on CT radiomics and clinical characteristics to predict the histologic grade of GC preoperatively, with AUCs of 0.752 and 0.793, respectively, in the training and validation cohorts [22]. In comparison to previous investigations, our radiomics model, which used both conventional polychromatic images and iodine-based MD images features, had higher AUCs of 0.900 and 0.851 in the training and testing cohorts, respectively. Our approach, on the other hand, coupled iodine-based MD images with conventional polychromatic images performed better than conventional polychromatic images alone. Delong’s test revealed no significant differences among these three models in the testing set, which might be attributed to the small number of patients in our research. The Brier score of model-CP–IMD showed better goodness of fit than model-CP, and decision curve analysis also showed that the model-CP–IMD was the optimal decision-making strategy to add the net benefit compared with model-CP. The better results were attributed to the use of iodine-based MD images, which have the potential to improve the depiction and characterization of hypoattenuating malignancies by increasing the contrast between a hypoattenuating lesion and normally enhancing parenchyma based on differences in tissue IC.

The vascular density and the blood volume in different tissue regions are reflected by IC. Vascular endothelial growth factor (VEGF) expression and microvessel density (MVD), both of which are directly related to the histological differentiation grade of GC, have been reported to be positively correlated with the IC value in previous research [15, 31–33]. Poorly differentiated tumors may increase vasopermeability and immature endothelial cells, explaining the high values of IC and MVD in poorly differentiated gastric cancer. Additionally, Li et al. demonstrated that the IC value was an independent predictor of lymph node metastasis in gastric cancer [34]. However, the measurement of IC in the previous study only reflected the average IC value in ROIs, and more information (such as tumor heterogeneity) was not evaluated in GC. Therefore, radiomic features of iodine-based MD images may provide more predictive information and improve the diagnosis. Li et al. developed a nomogram incorporating deep learning radiomics features extracted from 40-, 65-, and 100-keV images and CT-reported lymph node status. Their findings revealed that the nomogram not only performed well in predicting lymph node metastasis in gastric cancer but also significantly associated with patient’s prognosis [35]. This indicates that DESCT-based radiomics has great application prospects in gastric cancer. Previous studies have found that radiomic features derived from dual-energy CT iodine maps are useful in predicting breast cancer metastatic status [36] and identifying cervical lymph node metastases of thyroid cancer [37]. In this investigation, iodine-based MD images obtained from DESCT also performed well in predicting GC histologic grade, and the model’s performance was increased when paired with conventional polychromatic images. Furthermore, by combining radiomic and clinical signatures, our model was able to achieve even higher AUC. All of these demonstrated the importance of clinical and iodine-based radiomic features in GC histologic characteristics prediction.

The histological grade of GC was linked with age, sex, and tumor site, according to Huang et al. [22]. Similarly, Kim et al. [38] found that sex, age, and TNM stage are correlated with the histological classification of GC. Jing et al. [39] also revealed that some tumor makers, including CEA, and CA19-9, were significantly associated with pathological types and TNM staging. However, only the pT and pN stage showed significant differences between low- and high-grade GC in this investigation. Furthermore, pT and pN stage were meaningful and positively connected with tumor grade when it comes to model construction. It is worth noting that since this is a retrospective study, the stage here relates to the postoperative pathological information, but it is difficult for us to obtain totally precise staging information prior to surgery, predicting histologic grade using radiomic features may be more effective and successful.

Texture features are generally recognized as quantitative indicators of tumor heterogeneity because they are strongly related to the tumor microenvironment [40]. Our model-CP–IMD and model-Combine screened texture features mainly from wavelet and LoG filtered transformed images, with iodine-based MD images providing most of them. The findings showed that the features extracted from the iodine-based preprocessed images were more stable than those acquired from the original images. Our findings emphasize the value of using iodine-based MD images to extract high-order statistical features to assist in the radiological assessment and clinical decision-making.

There were certain limitations in our research. First, because this was a single-center retrospective study, selection bias was unavoidable. Second, the model was based on small sample size and there was no external validation. To evaluate the usefulness and robustness of this CP–IMD combined model, further research based on more patients and multiple centers is required. Third, only the venous phase images were employed to derive the radiomics features in this work. This was due to the fact that tumor differentiation from neighboring normal stomach tissue was at its peak in the portal venous phase, and additional phases should be investigated in the future.

In conclusion, our findings showed that DESCT-based radiomics models could help preoperatively predict histologic grade. The DESCT iodine-based MD images derived radiomic signatures have great potential for enhancing diagnostic performance. To fully comprehend the predictive potential of radiomics in this context, larger prospective studies are required.

## Data Availability

The dataset used in the current study was available from the corresponding author on reasonable request.
